# A systemic epithelial–immune–neural framework for pediatric atopic dermatitis–allergic rhinitis comorbidity

**DOI:** 10.3389/fimmu.2026.1864351

**Published:** 2026-08-07

**Authors:** Suning Mu, Yijie Wang, Wen Qin, Mengying Mao, Yan Wang, Yuqian Zhang, Youpeng Wang

**Affiliations:** 1 Heilongjiang University of Chinese Medicine, Harbin, China; 2 Second Affiliated Hospital of Heilongjiang University of Chinese Medicine, Harbin, China; 3 Guangde Hospital of Traditional Chinese Medicine, Guangde, Anhui, China

**Keywords:** allergic rhinitis, atopic dermatitis, children, comorbidity, cross-organ coupling, systemic epithelial–immune–neural framework

## Abstract

Atopic dermatitis (AD) and allergic rhinitis (AR) commonly coexist in childhood and are associated with an important clinical burden. Their frequent coexistence and substantial mechanistic overlap suggest that pediatric AD–AR comorbidity may involve patterns more complex than simple co-occurrence. However, existing models do not provide an operational approach for determining whether reproducible coupling between AD and AR disease activity extends beyond shared susceptibility and disease-specific responses occurring in parallel. In this Hypothesis and Theory article, we propose the systemic epithelial–immune–neural framework as an operational and falsifiable model for investigating pediatric AD–AR comorbidity. The framework integrates four interacting proposed mechanistic domains—epithelial barrier and alarmin signaling, type 2 immune activation, neuroimmune signaling, and microbiome regulation—and distinguishes established organ-specific mechanisms from candidate cross-organ coupling. It also separates core mechanisms from external inputs, contextual modifiers, and observable outcomes, and translates the proposed relationships into directed edges, feedback loops, testable predictions, and falsification criteria. Current evidence supports the biological plausibility of the framework and demonstrates substantial mechanistic overlap between AD and AR, but direct pediatric evidence for reproducible cross-organ coupling between AD and AR disease activity remains limited. The framework should therefore be regarded as a research model rather than a new diagnostic classification, composite network score, or separate treatment pathway. Clinical assessment and management should remain grounded in validated, guideline-based approaches for AD and AR. By organizing shared mechanisms and clinical observations into explicit and testable claims, the framework provides a structured basis for investigating the existence, potential mechanisms, heterogeneity, and clinical relevance of cross-organ coupling in pediatric AD–AR comorbidity.

## 1 Introduction

AD and AR are common inflammatory diseases of childhood and may occur sequentially or coexist. Pediatric AD–AR comorbidity is associated with a substantial overall clinical burden and warrants coordinated consideration of both diseases in affected children (1–3). However, the biological basis and organization of pediatric AD–AR comorbidity remain incompletely understood.

Existing chronological and mechanistic concepts provide important foundations for understanding allergic disease, including developmental progression, shared susceptibility, epithelial vulnerability, inflammatory endotypes, and interactions among barrier organs. However, these concepts do not by themselves determine whether pediatric AD–AR comorbidity primarily reflects shared susceptibility and disease-specific responses in AD and AR or also involves measurable cross-organ coupling.

To investigate this question, we propose the systemic epithelial–immune–neural framework as a hypothesis-driven, operational, and falsifiable research model. The framework is grounded in the mechanistic overlap between AD and AR across epithelial, immune, neural, and microbiome-related processes while distinguishing such overlap from candidate cross-organ coupling. It comprises four interacting but analytically distinguishable mechanistic domains: epithelial barrier and alarmin signaling, type 2 immune activation, neuroimmune signaling, and microbiome regulation.

In this Hypothesis and Theory article, we define the framework, specify its operational components, and distinguish it from related concepts. We then examine the epidemiological and clinical observations that motivate the model, evaluate evidence across the four mechanistic domains, and distinguish established organ-specific mechanisms from candidate cross-organ coupling. Finally, we consider implications for clinical assessment and management while separating current guideline-based practice from research biomarkers, unvalidated network profiling, and investigational cross-organ treatment effects. The aim is to provide a structured and revisable research model for investigating the existence, potential mechanisms, heterogeneity, and clinical relevance of cross-organ coupling in pediatric AD–AR comorbidity.

## 2 The systemic epithelial–immune–neural framework: operational definition, structure, and testable hypotheses

### 2.1 Operational definition and scope of the framework

We propose the systemic epithelial–immune–neural framework as a hypothesis-driven conceptual model for pediatric AD–AR comorbidity. The framework does not assume that AD and AR constitute a single disease, that their coexistence necessarily reflects direct biological interaction, or that the same mechanism operates in every affected child. Rather, it provides a structure for investigating whether, in at least a subset of children, organ-specific processes in the skin and nasal mucosa become measurably related through epithelial, immune, neural, and microbiome-related interactions.

Here, framework refers to an organizing structure that specifies the principal domains, nodes, proposed relationships, assumptions, and empirical consequences of the proposed model. Systemic does not imply generalized inflammation, identical pathology in the skin and nasal mucosa, or obligatory involvement of every proposed domain. It denotes the possibility that biologically or clinically relevant influences extend across tissues through candidate routes such as circulating epithelial-derived or immune mediators, trafficking or redistribution of activated immune cells, shared immune regulation, neuroimmune coordination, and microbiome-related modification of epithelial or immune thresholds.

Environmental exposure, age and immune maturation, genetic susceptibility, sensitization, infection, treatment, and selected behavioral processes are treated as external inputs, contextual modifiers, or mediators rather than as core mechanistic domains. These factors may initiate local disease activity, alter interactions among the core domains, or produce apparent covariation between AD and AR without direct cross-organ coupling. Their effects must therefore be considered when evaluating cross-organ coupling.

For the purposes of this framework, cross-organ coupling is operationally conceptualized as a reproducible temporal, biological, or clinical relationship between AD-related skin disease activity and AR-related nasal disease activity that remains detectable after major shared causes and confounders have been addressed. The co-occurrence of AD and AR alone is therefore insufficient to demonstrate cross-organ coupling. Evidence for cross-organ coupling would become progressively stronger if activity in one organ predicted subsequent changes in the other, if the relationship were mediated by measurable biological processes, if interaction-based models provided incremental predictive value beyond separate organ-specific models, or if perturbation of a candidate shared pathway produced reproducible changes in prespecified cross-organ outcomes. Contemporaneous symptom correlation without temporal ordering or adequate control of shared exposures would provide weaker evidence.

The framework organizes four proposed mechanistic domains: epithelial barrier and alarmin signaling, type 2 immune activation, neuroimmune signaling, and microbiome regulation. Their relative contributions may vary among children and across disease stages, allowing provisional research configurations such as barrier-predominant, type 2-high, neuroimmune-amplified, microbiome-perturbed, and mixed patterns.

Sleep disturbance, daytime fatigue or attention impairment, behavioral or emotional symptoms, impaired daily functioning, reduced health-related quality of life, and healthcare utilization are regarded primarily as observable system-level outcomes. They may participate in feedback relationships, but their causal position should be established empirically rather than assumed.

### 2.2 Distinction from existing concepts and the specific contribution of the framework

The proposed framework complements established models of allergic disease by organizing existing biological findings into testable claims about whether skin and nasal disease activity are measurably coupled in pediatric AD–AR comorbidity after shared determinants have been considered.

First, the framework differs from the classical atopic march. The atopic march describes a frequently observed developmental pattern in which AD precedes respiratory allergic disease. The present framework does not reject this trajectory but treats it as one possible pattern among several, including AR preceding AD, near-synchronous onset, fluctuating activity, or no fixed sequence. The distinction is therefore between a predominantly chronological model and a model that tests conditional cross-organ coupling (4).

Second, the framework differs from descriptive comorbidity, multimorbidity, and multiple-trajectory models. These approaches establish that allergic diseases may coexist or follow heterogeneous developmental courses, but they do not necessarily test whether activity in one organ retains predictive or biological information about subsequent activity in another after shared causes have been considered (5).

Third, epithelial barrier theory explains interface vulnerability, allergen penetration, tissue injury, and epithelial alarmin release in allergic diseases. The present framework retains epithelial barrier dysfunction as a core domain but examines it together with immune, neural, and microbiome-related processes in pediatric AD–AR comorbidity (6).

Fourth, type 2 inflammatory endotypes characterize dominant molecular or immunological programs within individual diseases. The proposed framework addresses a different question: whether epithelial, immune, neural, and microbiome-related processes contribute to candidate cross-organ coupling in pediatric AD–AR comorbidity (7).

Fifth, the skin–gut–lung axis and related epithelial-axis concepts emphasize distributed communication among barrier organs. The present framework is narrower in organ scope and clinical object: it focuses on candidate skin–nasal coupling in children with AD–AR comorbidity and links this question to specified nodes, directional edges, temporal predictions, and falsification criteria (8).

Sixth, existing neuroimmune models explain how sensory neurons, neuropeptides, inflammatory mediators, and immune cells contribute to pruritus or airway symptoms within individual tissues. The proposed framework extends these models by asking whether neuroimmune activity contributes to temporal coordination, amplification, or persistence across skin and nasal disease. This proposed cross-organ role remains to be demonstrated (9).

Taken together, the framework contributes five structural features: an operational definition of cross-organ coupling; specification of four core mechanistic domains; separation of core nodes, external inputs, contextual modifiers, mediators, and observable outcomes; distinction between evidence-supported within-organ edges and candidate cross-organ edges; and falsifiable hypotheses that can be evaluated through longitudinal observation, multidimensional profiling, and mechanistic perturbation.

### 2.3 Structural specification and operational components of the framework

This section specifies the framework’s core domains, representative research readouts, external inputs and contextual modifiers, observable outcomes, directed edges, and feedback loops.

#### 2.3.1 Core mechanistic domains and representative research readouts

Domain 1: epithelial barrier and alarmin signaling. Primary components include epithelial integrity and permeability, junctional-protein function, tissue injury and repair responses, and epithelial alarmin signaling in the skin and nasal mucosa. Representative research readouts may include skin transepidermal water loss; skin- or nasal-tissue expression of filaggrin and junction-related proteins; epithelial injury signatures; and tissue- or fluid-derived TSLP, IL-33, and IL-25 (10).

Domain 2: type 2 immune activation. Primary components include ILC2 activation, T helper 2 (Th2) cell polarization, eosinophil and mast-cell activity, type 2 cytokine signaling, and immunoglobulin E (IgE)-associated effector responses. Representative research readouts may include circulating or tissue eosinophil measures, IL-4, IL-5, IL-13, immune-cell phenotypes, and tissue inflammatory signatures (11). Allergen sensitization may provide relevant clinical context but should not be interpreted as a direct measure of cross-organ coupling.

Domain 3: neuroimmune signaling. Primary components include sensory-neuron activation or sensitization, IL-31-related signaling, neuropeptide release, immune–neural communication, pruritus, and sensory hypersensitivity. Representative research readouts may include IL-31, substance P, calcitonin gene-related peptide, itch ratings, objectively measured scratching or rubbing, nasal itch, and sensory-response measures (12).

Domain 4: microbiome regulation. Primary components include skin and nasal microbiome composition and function, microbiome perturbation, microbial metabolites, and microbiome-associated epithelial and immune responses.

Within the nasal ecosystem, anterior-nares samples should be distinguished operationally from nasal mucosal samples used to characterize AR-related mucosal activity, because these sites represent related but non-interchangeable microbial niches. In this framework, the anterior nares are considered both a local nasal microbial niche and a potential reservoir interface for organisms shared with the skin, particularly *Staphylococcus aureus*. Anterior-nares carriage alone is therefore neither a direct measure of AR mucosal inflammation nor evidence of skin–nasal coupling.

Representative research readouts may include microbial diversity, community composition, the abundance of relevant organisms such as *S. aureus*, paired, site-specific sampling of the skin, anterior nares, and nasal mucosal sites, functional metagenomic profiles, strain-level features when available, and selected microbial metabolites (13).

The four domains are analytically distinguishable but biologically interconnected. Their relative contributions may vary among children and across disease stages, and no single readout is expected to capture the complete state of the framework.

#### 2.3.2 External inputs, contextual modifiers, mediators, and observable outcomes

External inputs include allergens, irritants, pollutants, climate and season, infection, and antibiotic exposure. Host and contextual modifiers include age, developmental and immune maturation, genetic susceptibility, sensitization, baseline disease severity, and prior treatment history. Treatment exposure, adherence, scratching, rubbing, and other symptom-related behaviors may function as mediators or modifiers depending on the causal question being examined.

These factors may act on one or both organs, alter activation thresholds or interactions among the four domains, or produce apparent covariation between AD and AR without direct cross-organ coupling. Shared environmental exposure should therefore be represented as a parallel input to the skin and nasal mucosa rather than as a pathway transmitting disease from one organ to the other.

Observable outcomes include AD severity and control, AR symptom burden and control, pruritus, nasal obstruction, sleep disturbance, daytime fatigue or attention impairment, behavioral or emotional symptoms, daily functioning, health-related quality of life, and healthcare utilization. These outcomes should not automatically be classified as mechanistic nodes. Their possible feedback effects require temporal or interventional evidence.

#### 2.3.3 Directed edges and directionality

Edges represent proposed directional relationships through which a change in one node is expected or hypothesized to alter the state of another. The framework distinguishes between organ-specific relationships supported to varying degrees by existing evidence and candidate cross-organ coupling routes requiring direct validation.

The principal within-organ relationships can be summarized as follows:

1. Epithelial ↔ immune: epithelial injury and alarmin signaling activate immune pathways, whereas immune mediators may impair epithelial integrity or modify barrier repair.
2. Immune ↔ neuroimmune: immune mediators may activate or sensitize sensory pathways, whereas neural mediators may modify immune-cell activity.
3. Epithelial ↔ neuroimmune: epithelial injury and alarmin signaling may activate or sensitize sensory pathways, whereas neural mediators, neurogenic inflammation, and sensory-driven scratching or rubbing may directly or indirectly affect epithelial integrity and repair.
4. Microbiome ↔ epithelial: microbiome composition, function, or perturbation may influence epithelial integrity and repair, whereas epithelial conditions may reshape the local microbial environment.
5. Microbiome ↔ immune: microbial communities and their products may modify local immune activity, whereas immune-mediated changes in the tissue environment may in turn influence microbial composition and function.

The strength and direction of these reciprocal relationships may vary by tissue, disease stage, treatment, and sampling context.

Candidate routes relevant to cross-organ coupling include:

1. Skin epithelial or immune activity → circulating mediators or immune-cell redistribution → nasal epithelial or immune activity, with the reverse direction also considered;
2. Skin or nasal neuroimmune activity → shared sensory, autonomic, central, or behavioral processes → activity or symptom expression in the other organ;
3. Microbiome state as a potential modifier of the strength, threshold, or persistence of epithelial–immune–neural relationships across organs;
4. Anterior-nares microbial carriage as a potential reservoir interface between the nasal and cutaneous ecosystems, particularly for organisms such as *S. aureus* that may be detected at both sites.

The third route is a modulatory hypothesis and does not require direct microbial transfer between the skin and nasal mucosa. The fourth route distinguishes the local nasal ecological role of the anterior nares from their proposed reservoir role in skin–nasal connectivity. Evidence for the latter would require site-resolved longitudinal data showing that anterior-nares microbial or strain-level patterns precede or predict subsequent cutaneous colonization, AD activity, or coordinated skin–nasal changes after relevant local and shared determinants have been addressed. Concurrent detection of the same organism in the anterior nares and skin would indicate shared carriage, but would not by itself establish directionality or cross-organ coupling.

In the framework and its figures:

A ↔ B denotes a potentially reciprocal relationship, although the evidence need not be equally strong in both directions.

A → B denotes a proposed directional edge requiring direct validation.

Candidate cross-organ edges represent testable hypotheses rather than established causal pathways. Their direction, mediators, effect size, and reproducibility should be examined through longitudinal, mediation, or interventional studies.

#### 2.3.4 Feedback loops

The framework specifies four principal feedback loops. The first three have varying degrees of organ-specific mechanistic support, whereas the fourth is a candidate cross-organ loop proposed for pediatric AD–AR comorbidity.

Loop 1: epithelial barrier–type 2 amplification loop

Epithelial injury or barrier dysfunction → alarmin release → type 2 immune activation → cytokine-mediated barrier impairment or altered repair → further epithelial vulnerability.

Loop 2: neuroimmune sensory–behavioral amplification loop

Inflammatory mediators → sensory-neuron activation or sensitization → pruritus → scratching → mechanical epithelial injury → renewed epithelial and immune activation.

This loop is best established as the itch–scratch cycle in AD. Nasal itch, rubbing, sneezing, and sensory hyperresponsiveness may represent related upper-airway amplification processes, but they should not be assumed to reproduce the cutaneous loop in an identical form.

Loop 3: microbiome–epithelial barrier–immune feedback loop

Microbiome perturbation or altered microbial function may impair epithelial barrier integrity and promote local inflammatory activation. Barrier injury and immune-mediated changes in the tissue environment may in turn favor microbial instability or pathobiont dominance, thereby sustaining a microbiome–epithelial barrier–immune amplification loop. Conversely, a relatively stable and functionally balanced microbiome may help maintain epithelial barrier integrity and local immune homeostasis.

Loop 4: candidate cross-organ amplification loop

Disease activity in one organ → changes in circulating mediators, immune-cell distribution, neuroimmune susceptibility, or coupling thresholds → altered activity or symptom expression in the other organ → changes in system-level burden that may subsequently modify overall disease activity.

The fourth loop does not assume a fixed skin-to-nose-to-skin sequence. It may operate in either direction, may be bidirectional or stage-dependent, and may occur only in particular patient subgroups rather than in every child. Its mediators, effect size, and reproducibility remain to be determined.

Feedback within the framework is not assumed to be uniformly pathogenic. Epithelial repair, regulatory immune activity, a relatively stable microbiome, treatment, and reduction of clinically relevant exposure may weaken amplification and promote stability.

Accordingly, the first three loops are supported mainly by organ-specific evidence, whereas the fourth cross-organ loop remains a testable hypothesis requiring longitudinal or interventional validation.

### 2.4 Core falsifiable hypotheses, testable predictions, and falsification criteria

The framework is organized around three core hypotheses. They represent the principal relational or causal claims of the model, whereas the predictions specify their observable empirical consequences. A single negative study would not necessarily falsify a hypothesis, but consistent failure of the specified predictions across adequately powered, longitudinal, and independently replicated studies would require revision or rejection.

#### Core hypothesis 1: residual cross-organ coupling

After major shared causes and confounders are accounted for, a subset of children with AD–AR comorbidity will show reproducible temporal or biological dependence between skin and nasal disease activity.

##### Prediction 1: skin and nasal disease activity exhibits residual temporal coupling

Repeated longitudinal assessment should test whether changes or flares in AD precede measurable changes in AR symptoms or nasal inflammation and whether nasal disease activity similarly predicts subsequent skin activity, with adjustment for age, season, sensitization, infection, shared exposure, treatment, baseline severity, and observation frequency.

Operationally, residual coupling should be evaluated using longitudinal models selected according to the sampling design to estimate within-child, time-lagged associations while appropriately addressing stable between-child differences, within-child autocorrelation, unequal observation intervals, and time-varying confounding. Random-intercept cross-lagged panel models may be suitable for scheduled repeated assessments, whereas multilevel vector autoregressive or dynamic structural equation models may be considered for intensive longitudinal data.

Support would require reproducible time-lagged associations and improved prediction beyond separate organ-specific models; the prediction would be weakened if apparent covariation were explained by shared exposure, atopic susceptibility, surveillance patterns, seasonality, or concurrent treatment.

##### Prediction 2: epithelial alarmin profiles provide incremental information about subsequent AR risk or activity

Prospective cohorts of children with AD should test whether skin-, nasal-, or blood-based epithelial alarmins, including TSLP and IL-33, predict subsequent AR development or activity beyond established clinical predictors. Support would require incremental predictive value in independent cohorts; the prediction would be weakened if the markers reflected only concurrent AD severity or added no information beyond AD severity, sensitization, family history, age, treatment, and environmental exposure.

##### Prediction 3: neuroimmune signatures predict cross-organ symptom coupling

Longitudinal studies should assess whether neuroimmune measures—including IL-31, neuropeptides, and sensory-response measures—predict synchronous or reproducibly time-lagged changes in skin and nasal disease activity, and whether symptom-related behaviors or sleep disturbance mediate or modify these associations.

The prediction would be supported if neuroimmune measures improved prediction beyond conventional organ-specific severity and inflammatory measures. It would be weakened if associations disappeared after adjustment for local disease severity or if the measures predicted subjective symptoms without predicting objective changes in either organ.

#### Core hypothesis 2: heterogeneous and developmentally variable configurations

Where cross-organ coupling is present, its pattern and strength will vary among children and across developmental or disease stages because different combinations of epithelial, immune, neural, microbiome-related, environmental, and behavioral processes may predominate.

##### Prediction 4: pediatric AD–AR comorbidity contains reproducible multidimensional subgroups

Integrated epithelial, immune, neural, microbiome, clinical, and exposure-related measurements should test whether children with similar conventional diagnoses differ in dominant configurations of the proposed framework.

The prediction would be supported if independently reproducible subgroups differed in disease trajectory, symptom pattern, relapse risk, system-level burden, or treatment response and provided information beyond conventional severity scores. It would be weakened if clustering produced unstable groups or only simple gradients of overall disease severity.

##### Prediction 5: microbiome-related features modify the strength or expression of cross-organ coupling

Paired longitudinal studies of the skin, nasal cavity, and anterior nares should test whether microbial composition, microbial function, strain-level features, or anterior-nares reservoir status are associated with disease trajectories and modify relationships among epithelial, immune, and clinical measures across organs. Particular attention should be given to whether anterior-nares reservoir status, especially *S. aureus* carriage, and skin–nasal microbial or strain-level concordance precede recurrent skin colonization, AD flares, AR activity, or coordinated changes in skin and nasal symptoms.

The prediction would be supported if microbiome-related measures reproducibly improved prediction of disease trajectory, relapse, or cross-organ coupling beyond local severity, treatment, antibiotic exposure, infection, season, environmental exposure, and sampling site. It would be strengthened further if longitudinal functional or strain-level data showed temporal ordering consistent with a reservoir, modifier, or mediator role. It would be weakened if microbial variation were consistently explained by local disease activity, external confounders, or sampling artifacts, or provided no incremental information beyond established organ-specific models.

#### Core hypothesis 3: mechanistic perturbation of candidate shared pathways

If a candidate shared pathway contributes causally to cross-organ coupling, modification of that pathway should alter at least one prespecified cross-organ readout or the temporal relationship between organ-specific outcomes, beyond changes explained by concomitant treatment, shared exposure, seasonality, natural fluctuation, or regression to the mean, or parallel direct pharmacological effects of the intervention in both tissues.

##### Prediction 6: pathway-directed treatment can serve as a mechanistic perturbation test

Prospective studies or appropriately designed secondary analyses of children receiving pathway-directed treatment for an established disease indication should test whether modification of type 2 or neuroimmune pathways is accompanied by prespecified outcomes beyond the treated organ, including changes in skin or nasal activity, candidate shared mediators, or temporal coupling. Studies should prespecify the treated disease, approved indication, age group, concomitant therapy, outcomes, follow-up duration, and major confounders.

Because systemic therapies may directly engage the same pathway in both skin and nasal tissues, concurrent improvement in the two organs cannot by itself distinguish cross-organ mediation from parallel tissue-specific drug effects. Future studies should therefore prespecify a causal model and combine repeated organ-specific outcomes with measures of local pathway activity and candidate circulating or cellular mediators. Stronger evidence for cross-organ mediation would require a temporally ordered indirect pathway in which treatment-related change in one organ or in a shared mediator predicts subsequent change in the other after direct pathway engagement in that organ has been considered. When suitable comparison data are available, contrasts with organ-directed treatment or treatments acting through different pathways, together with longitudinal causal mediation analysis under explicit assumptions, may help separate direct and indirect effects.

Support would require reproducible cross-organ changes with appropriate comparators, temporal consistency, and evidence of a prespecified indirect pathway beyond direct drug action in each tissue; concurrent improvement alone would be insufficient. The prediction would be weakened if apparent cross-organ improvement were fully explained by parallel tissue-specific effects, concomitant care, seasonal change, shared exposure, or natural fluctuation.

Each core hypothesis has a distinct falsification criterion. The first would be weakened if longitudinal studies consistently failed to identify residual cross-organ dependence after adjustment for shared causes. The second would be weakened if multidimensional analyses repeatedly failed to identify reproducible heterogeneity beyond conventional severity gradients. The third would be weakened if appropriately controlled mechanistic perturbation studies consistently showed no change in outcomes or coupling measures beyond the disease for which treatment was initiated, or if apparent cross-organ improvement was fully attributable to parallel direct drug effects in both tissues.

## 3 Epidemiology and disease burden

AD–AR comorbidity is common in children, and its co-occurrence may exceed that expected by chance (14, 15). A systematic review and meta-analysis reported a pooled rhinitis prevalence of 40.5% among individuals with AD and an OR of 3.25 for the association between AD and AR (16). A subsequent global mixed-age analysis estimated the prevalence of AR among individuals with AD at 41.4% and reported an overall OR of 2.42 for the AD–AR association (17). Pediatric studies further link AD with concurrent or subsequent AR (18), with higher risks reported for early-onset, persistent, or more severe AD and for early-life AD accompanied by food allergy (19–21). AR prevalence also increases across AD severity categories and with age at AD diagnosis (22, 23). Conversely, AD was reported in 19.9%–25.7% of pediatric AR cohorts (24, 25). Population-based studies additionally identified age-related variation in concurrent AD and AR (26). Importantly, longitudinal analyses showed that very early-onset persistent AD remained associated with subsequent hay fever after adjustment for relevant measured covariates (27). These findings are summarized in **Table 1**.

**Table 1:** Epidemiological studies relevant to pediatric AD–AR comorbidity.

Study	Population/sample	Design	Major findings relevant to AD–AR
Hong S et al. (2012)	31,201 Korean children	Cross-sectional survey	Concurrent AD and AR were reported in 8.7% of participants (14).
Pinart M et al. (2014)	10,107 children assessed at ages 4 and 8 years	Population-based cohort study	The co-occurrence of eczema/AD and AR exceeded that expected by chance (15).
Knudgaard MH et al. (2021)	341 studies in the qualitative analysis and 302 in the quantitative analysis	Systematic review and meta-analysis	The pooled prevalence of rhinitis among patients with AD was 40.5%; AD was associated with AR (OR 3.25, 95% CI 2.26–4.66) (16).
Liu Y et al. (2026)	278 studies; global mixed-age populations	Systematic review with hierarchical Bayesian modeling	Among individuals with AD, estimated prevalence was 45.1% for rhinitis and 41.4% for AR; the overall OR for AR was 2.42 (17).
Sultész M et al. (2020)	3,836 schoolchildren	Cross-sectional study	Eczema was associated with AR (OR 1.899, 95% CI 1.568–2.300; p < 0.0001) (18).
von Kobyletzki LB et al. (2012)	3,124 children aged 1–2 years	Prospective cohort study	Early childhood eczema was associated with an approximately threefold higher risk of rhinitis 5 years later; earlier onset, persistence, and greater severity were associated with higher risk (19).
Schoos AMM et al. (2022)	368 children from an at-risk mother–child cohort	Longitudinal cohort study	Early-onset AD was associated with childhood AR (GEE OR 1.56, 95% CI 1.01–2.41); increasing early-onset AD severity was also associated with AR (GEE OR 1.09, 95% CI 1.05–1.13) (20).
Jung S et al. (2022)	1,579 children	Prospective cohort study	AD with coexisting food allergy in early life was strongly associated with AR in schoolchildren (21).
Silverberg JI et al. (2021)	7,465 children from 18 countries	Cross-sectional survey	AR prevalence increased across mild, moderate, and severe AD categories (41.3%, 50.2%, and 52.2%, respectively) (22).
Leshem YA et al. (2025)	177,081 pediatric patients with AD	Retrospective real-world cohort study	AR prevalence at AD diagnosis increased with age, from 2.2% in children aged <3 years to 23.2% in those aged 12–17 years (23).
Fritzsching B et al. (2023)	11,036 children with AR initiating allergen immunotherapy	Retrospective real-world study	AD was present in 19.9% of the pediatric AR/AIT cohort (24).
Serbes M. (2023)	874 children with AR	Retrospective medical-record review	AD was present in 25.7% of children with AR (25).
Kim J et al. (2025)	Korean children aged 6–7, 9–10, and 12–13 years; repeated surveys, 1995–2022	Repeated cross-sectional population study	In 12–13-year-olds, the prevalence of AD with rhinitis increased from 1995 to 2022 (p for trend < 0.001); the study did not estimate AD prevalence within an AR cohort (26).
Miltner LA et al. (2024)	6,852 children and adolescents	Population-based cohort study	After adjustment for relevant measured covariates, persistent childhood eczema—especially the very early-onset persistent phenotype—was associated with a higher risk of subsequent hay fever (27).

This table summarizes epidemiological evidence relevant to pediatric AD–AR comorbidity, including co-occurrence, bidirectional prevalence, age- and severity-related patterns, longitudinal associations, and findings after adjustment for relevant measured covariates. Original study terminology is retained for study-specific estimates because AD-related outcomes may be reported as eczema or atopic eczema, whereas AR-related outcomes may be defined as rhinitis, allergic rhinitis, rhinoconjunctivitis, or hay fever. These terms are not necessarily diagnostically equivalent; otherwise, AD and AR are used throughout for consistency.

These epidemiological associations may reflect shared susceptibility, common environmental exposures, sequential disease development, and/or candidate cross-organ coupling; epidemiological data alone cannot distinguish among these explanations. Early-life and environmental factors may influence the risk and longitudinal trajectories of both AD and AR and should therefore be considered as shared determinants when their association is evaluated (28).

This frequent comorbidity is associated with a substantial combined burden, including higher healthcare costs, more outpatient visits and hospitalizations, and greater use of biologic therapies (29), as well as sleep disturbance, daytime fatigue, impaired attention, and increased risks of psychological and behavioral problems, including anxiety and depression (30, 31). These findings support coordinated clinical assessment of pediatric AD–AR comorbidity but do not, by themselves, demonstrate cross-organ coupling.

## 4 Clinical interaction patterns in pediatric AD–AR comorbidity: observed associations and testable cross-organ hypotheses

### 4.1 Longitudinal trajectories: variability in the order of clinical onset

The order of clinical onset between AD and AR varies among children. As summarized in Section 3 and **Table 1**, AD often precedes AR, although AR-first and near-synchronous trajectories are also observed. Within the proposed framework, this heterogeneity may reflect differences in the timing at which organ-specific processes in the skin and nasal mucosa reach thresholds for clinical expression. These trajectories are compatible with the framework but do not themselves demonstrate cross-organ coupling. Repeated organ-specific assessments and paired biomarker measurements are needed to test whether activity in one organ predicts subsequent activity in the other after major shared determinants and confounders have been addressed.

### 4.2 Horizontal interactions: concurrent clinical activity and candidate bidirectional relationships

The frequent coexistence of AD and AR and the association between greater AD severity and later AR risk are summarized in **Table 1**. The proposed systemic epithelial–immune–neural framework offers one hypothesis for interpreting these associations. Shared epithelial barrier vulnerability, type 2 immune activation, and neuroimmune signaling may create a biological context in which skin and nasal disease activity becomes partially coordinated.

However, specific bidirectional cross-organ coupling remains unproven. In the AD-to-AR direction, whether scratching-related neuroimmune activity predicts subsequent changes in AR symptoms or nasal inflammation has not been established. In the AR-to-AD direction, nasal obstruction and sleep disruption may increase the overall symptom burden, while nasal discharge and repeated wiping or rubbing may cause local irritation.

### 4.3 System-level clinical outcomes and potential feedback relationships

**Figure 1:**
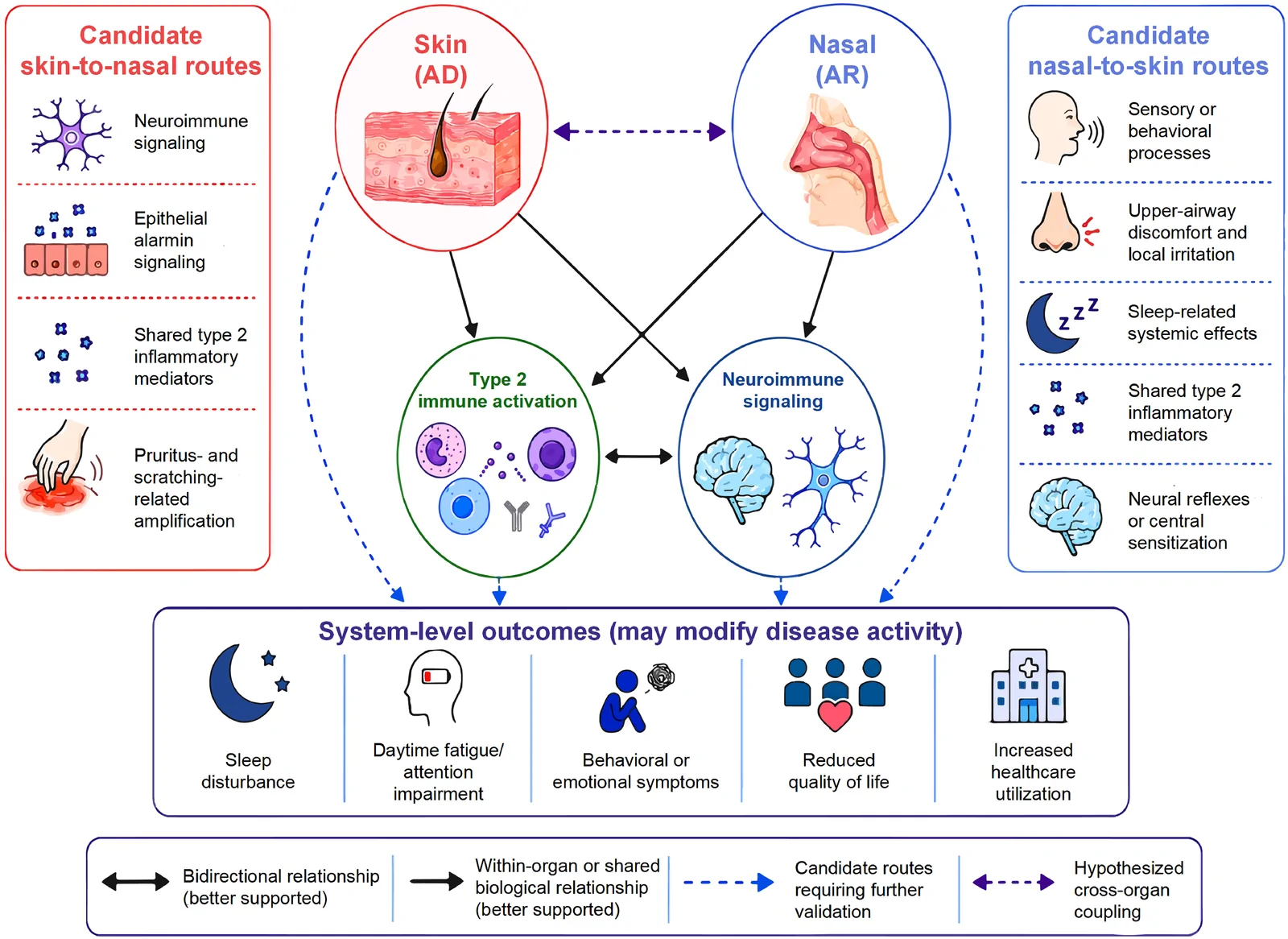
Clinical interaction patterns and candidate cross-organ coupling in pediatric AD–AR comorbidity. The figure summarizes proposed relationships between skin disease activity in AD and nasal mucosal disease activity in AR. Type 2 immune activation and neuroimmune signaling are shown as shared processes that may contribute to organ-specific amplification and candidate cross-organ coupling. The side panels present candidate skin-to-nasal and nasal-to-skin pathways, whereas system-level outcomes include sleep disturbance, daytime fatigue or attention impairment, behavioral or emotional symptoms, reduced quality of life, and increased healthcare utilization. Black double-headed arrows indicate relatively well-supported bidirectional relationships; black single-headed arrows indicate relatively well-supported biological relationships between organ-specific disease activity and shared mechanistic processes; blue dashed arrows indicate candidate routes requiring further validation; and the purple dashed double-headed arrow indicates hypothesized cross-organ coupling.

As described in Section 3, pediatric AD–AR comorbidity is associated with broader clinical consequences, particularly sleep disturbance and impaired daytime, behavioral, and emotional functioning. Within the proposed framework, these consequences are regarded primarily as system-level outcomes rather than evidence of cross-organ coupling. Sleep disruption may subsequently influence symptom perception, scratching or rubbing behavior, treatment adherence, and daily functioning, but any feedback effect on later AD or AR activity remains hypothetical. **Figure 1** summarizes these candidate clinical pathways, shared type 2 immune and neuroimmune processes, and associated system-level outcomes.

## 5 Mechanistic evidence supporting the proposed framework: established organ-specific pathways and candidate cross-organ coupling

This section examines biological evidence relevant to the four proposed mechanistic domains and evaluates whether that evidence supports cross-organ coupling between AD and AR disease activity. To avoid equating biological similarity with cross-organ coupling, the discussion distinguishes established organ-specific mechanisms, processes shared by AD and AR, and candidate cross-organ coupling that remain to be validated.

### 5.1 Mechanistic evidence across the four core domains

#### 5.1.1 Epithelial barrier and alarmin signaling: local evidence and candidate cross-organ implications

The skin and nasal mucosa are anatomically and functionally distinct epithelial interfaces, but both participate in protection against environmental agents. In AD, impaired epidermal differentiation, junctional integrity, and barrier function facilitate water loss, penetration of irritants or allergens, and local inflammation. Loss-of-function variants in filaggrin and other barrier-related abnormalities provide strong evidence for epithelial susceptibility in AD and are associated with an increased risk of respiratory allergic disease in selected populations (32). In AR, the nasal epithelium may exhibit impaired junctional integrity and increased permeability. Histamine and type 2 inflammatory cytokines can further disrupt nasal epithelial barrier function, facilitating allergen penetration and local inflammatory amplification (33).

Epithelial injury can induce alarmins such as TSLP, IL-33, and IL-25, which promote local activation of dendritic cells, ILC2s, and type 2 immune responses in the skin or respiratory mucosa (34–37). Current evidence supports local epithelial dysfunction and shared alarmin biology in AD and AR. However, direct evidence that epithelial activation in the skin induces subsequent nasal epithelial or inflammatory changes, or the reverse, remains limited in children with AD–AR comorbidity.

#### 5.1.2 Type 2 immune activation: substantial mechanistic overlap across AD and AR

Type 2 immune activation is a central feature of both AD and AR. Epithelial alarmins promote ILC2 activation and Th2 polarization, while IL-4, IL-5, and IL-13 collectively contribute to IgE-associated responses, eosinophilic inflammation, mucus production, pruritus, and epithelial barrier dysfunction (7, 11, 37, 38). Mast cells, eosinophils, Th2 cells, ILC2s, and their associated cytokines therefore represent an important area of mechanistic overlap between the two diseases.

Circulating mediators and immune-cell redistribution have been proposed as candidate routes through which allergic immune activity might extend beyond a single tissue. However, similar cytokines or effector-cell populations in AD and AR do not demonstrate cross-organ coupling. Whether type 2 pathways directly mediate skin–nasal coupling in children with both diseases remains to be tested through paired longitudinal or perturbation-based studies.

#### 5.1.3 Neuroimmune signaling: local amplification and candidate cross-organ coordination

Neuroimmune signaling links inflammatory activity to pruritus, nasal itching, sneezing, irritation, and other sensory symptoms. In AD, IL-31 activates IL-31-responsive sensory neurons, including TRPV1- and TRPA1-expressing subsets, thereby contributing to pruritus, scratching, and the itch-scratch cycle (39–41). Experimental studies also suggest that allergen-induced sensory-neuron activation and substance P release can influence dendritic-cell migration and type 2 immune responses (42). In AR, nasal sensory-afferent activation contributes to nasal itching, sneezing, and reflex secretory responses, and neuropeptides such as substance P and CGRP may modulate local immune, vascular, epithelial, and glandular responses (43, 44).

Potential cross-organ routes include shared autonomic or central regulation, altered sensory responsiveness, and symptom-related behaviors such as scratching and rubbing. These mechanisms are biologically plausible, but current evidence mainly supports local neuroimmune amplification within AD and AR separately. Their contribution to synchronized flares, time-lagged changes, or persistent skin–nasal coupling remains to be tested.

#### 5.1.4 Microbiome regulation: local mechanisms and candidate skin–nasal microbial connectivity

Importantly, microbiome regulation in this framework does not refer only to microbial transfer between organs, but also includes microbial effects on epithelial integrity, immune thresholds, and inflammatory persistence. Microbiome composition and function are shaped by age, immune maturation, infection, antibiotic exposure, environmental conditions, host epithelial characteristics, treatment exposure, and sampling site. Because microbial colonization and immune development occur concurrently in early life, microbiome perturbation may influence allergic susceptibility by modifying epithelial barrier integrity, innate immune tone, type 2 inflammatory thresholds, and host–microbial tolerance (45).

In AD, skin microbiome perturbation is associated with epithelial barrier dysfunction, inflammatory activity, pruritus, and disease severity, with *S. aureus* representing the best-characterized pathobiont. In AR, alterations in the nasal microbiome have also been associated with mucosal inflammation and disease activity. These findings support local microbiome–epithelial–immune relationships in both diseases, although most available studies remain observational and cannot reliably determine whether microbial changes are causes, amplifiers, consequences, or biomarkers of disease activity (46–49).

A candidate microbial connection between the nasal and cutaneous ecosystems may involve the anterior nares. The anterior nares form part of the nasal microbial ecosystem, but samples from this site should not be treated as interchangeable with nasal mucosal samples used to assess AR-related mucosal activity. Their relevance to the framework is therefore twofold. First, anterior-nares microbial communities may reflect local nasal ecological conditions. Second, the anterior nares may serve as a potential reservoir interface for organisms shared with the skin, particularly *S. aureus*. These roles should be analyzed separately: anterior-nares carriage may be associated with local nasal status without contributing to cross-organ coupling, whereas a reservoir role would imply a reproducible temporal or strain-level relationship with subsequent cutaneous colonization or disease activity.

Accordingly, cross-sectional concordance between skin and anterior-nares microbiota is insufficient to establish directionality or biological coupling. The reservoir hypothesis should be tested using paired, site-resolved longitudinal sampling of skin, anterior nares, and nasal mucosal sites relevant to AR, with strain-level resolution where feasible. Support would be strengthened if changes in anterior-nares carriage preceded recurrent skin colonization, AD flares, or coordinated skin–nasal disease activity after adjustment for local disease severity, infection, antibiotic exposure, treatment, season, environmental exposure, and sampling site (47, 50).

Microbial functional outputs may influence AD and AR through barrier- and immune-related mechanisms that are not fully captured by taxonomic composition alone. In the skin and nasal mucosa, microbial proteases, toxins, or superantigen-related effects may damage epithelial junctions, impair epithelial repair, and promote epithelial alarmin release. Microbial metabolites and antimicrobial-peptide interactions may alter local antimicrobial defense, innate immune tone, and microbial niche stability. Quorum-dependent behavior and biofilm-associated states may sustain pathobiont dominance or persistent colonization, whereas strain-level variation may explain why the same microbial taxon has different inflammatory potential across children. Through these mechanisms, functional microbial changes may lower epithelial activation thresholds, amplify local epithelial–immune loops, and contribute to disease persistence, flare susceptibility, or coordinated changes in skin and nasal disease activity.

Future studies should therefore combine paired skin, nasal, and anterior-nares sampling with functional or strain-level profiling, while controlling for infection, antibiotic exposure, topical and intranasal treatment, season, sampling site, environmental exposure, and disease severity. Until such evidence is available, microbiome regulation should be interpreted as a local disease modifier and candidate cross-organ microbial contributor rather than as an established mechanism of skin–nasal coupling (46, 47, 50, 51).

### 5.2 Integrated mechanistic interpretation and remaining evidence gaps

Within each organ, epithelial barrier and alarmin signaling, type 2 immune activation, neuroimmune signaling, and microbiome-related regulation interact and may contribute to disease persistence, amplification, and flare susceptibility. These local mechanisms provide a biologically plausible basis for considering skin–nasal relationships in pediatric AD–AR comorbidity, with candidate links including circulating mediators, immune-cell redistribution, shared type 2 susceptibility, neuroimmune regulation, and microbiome-related modification of epithelial or immune activation thresholds.

**Figure 2:**
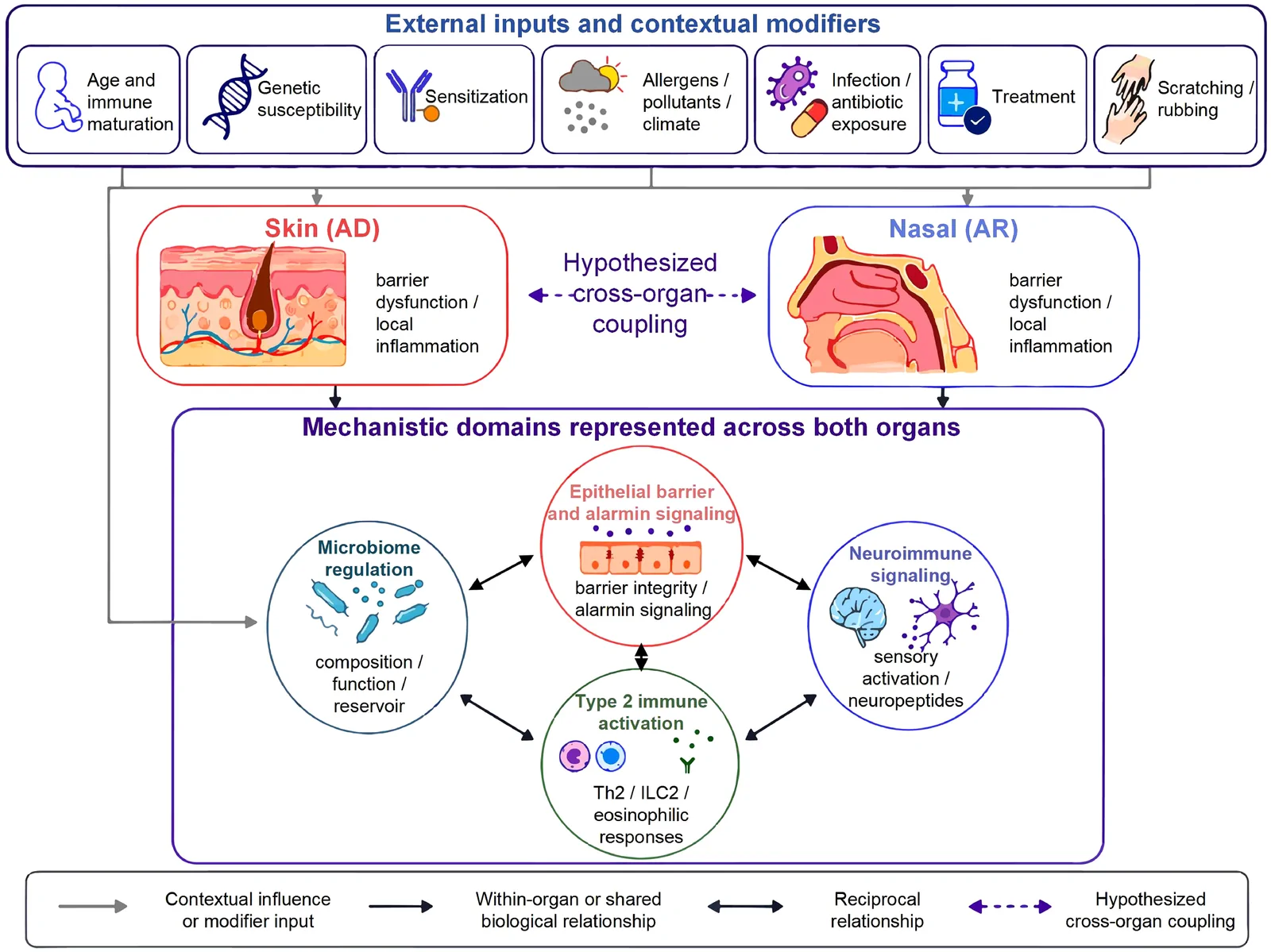
Hypothesized mechanistic relationships in pediatric AD–AR comorbidity. The image illustrates the central hypothesis of the systemic epithelial–immune–neural framework: in a subset of children with AD–AR comorbidity, AD-related skin activity and AR-related nasal mucosal activity may become measurably coupled through four interacting domains: epithelial barrier and alarmin signaling, type 2 immune activation, neuroimmune signaling, and microbiome regulation. External inputs and contextual modifiers may influence one or both organs and alter activation thresholds among these domains. Gray arrows indicate contextual influences or modifier inputs; black single-headed arrows indicate within-organ or shared biological relationships; black double-headed arrows indicate reciprocal relationships; and the purple dashed double-headed arrow indicates hypothesized cross-organ coupling.

However, shared pathways or parallel abnormalities in AD and AR do not by themselves establish the direction, magnitude, temporal ordering, or causal nature of cross-organ coupling. Current evidence mainly supports these mechanisms within AD or AR considered separately, whereas direct evidence of reproducible skin–nasal coupling in children with both diseases remains limited. **Table 2** summarizes the operational components, evidence-source stratification and pediatric relevance, interpretation for pediatric AD–AR coupling, and remaining research gaps across the four mechanistic domains. These proposed mechanistic relationships, together with the relevant external inputs and contextual modifiers, are summarized in **Figure 2**.

**Table 2:** Operational components and narrative evidence appraisal of the four core mechanistic domains in the proposed framework.

Core mechanistic domain	Operational components and representative research readouts	Evidence base in AD and AR	Evidence-source stratification and pediatric relevance	Interpretation for pediatric AD–AR coupling	Narrative support for domain inclusion and principal gap
Epithelial barrier and alarmin signaling	Epithelial integrity and permeability; junctional proteins; tissue injury and repair; TSLP, IL-33, and IL-25. Representative readouts include skin transepidermal water loss; skin- or nasal-tissue expression of barrier- and junction-related proteins; epithelial injury signatures; and tissue- or fluid-derived alarmins.	AD: strong evidence for epidermal barrier dysfunction, filaggrin-related susceptibility, and epithelial alarmin signaling. AR: evidence for impaired nasal epithelial junctional integrity, increased permeability, and cytokine-associated barrier dysfunction.	Direct pediatric AD–AR: Very limited; no paired longitudinal evidence of skin–nasal epithelial coupling. Adult/mixed-age human: Supports epidermal barrier dysfunction in AD, nasal epithelial barrier dysfunction in AR, and epithelial alarmin pathways across both diseases. Animal/experimental: Supports IL-25-, IL-33-, and TSLP-driven epithelial–type 2 immune responses. Pediatric single-organ AD/AR: Strong evidence for skin-barrier dysfunction in pediatric AD; separate pediatric AR studies support nasal epithelial abnormalities.	Direct paired evidence showing that epithelial activation in one organ predicts subsequent epithelial or inflammatory changes in the other remains limited.	Narrative grade: Moderate Principal gap: Longitudinal paired skin and nasal epithelial measurements, with temporal assessment of alarmins and organ-specific disease activity (34–37).
Type 2 immune activation	ILC2 and Th2 activation; eosinophil and mast-cell activity; IL-4, IL-5, and IL-13 signaling; IgE-associated effector responses. Representative readouts include blood or tissue eosinophils, type 2 cytokines, immune-cell phenotypes, and tissue inflammatory or transcriptomic signatures.	Type 2 inflammation is a well-established component of both AD and AR, with substantial overlap in cytokines, effector-cell populations, and epithelial–immune interactions.	Direct pediatric AD–AR: Very limited; direct immune-mediated skin–nasal coupling has not been demonstrated. Adult/mixed-age human: Supports shared IL-4, IL-5, IL-13, IgE, and eosinophilic pathways. Animal/experimental: Supports alarmin–ILC2–Th2 amplification and type 2 cytokine effects. Pediatric single-organ AD/AR: Strong pediatric evidence in both AD and AR separately.	Shared type 2 biology has not been shown to constitute direct immune transmission or reproducible skin–nasal coupling in children with both diseases.	Narrative grade: Relatively strong Principal gap: Paired longitudinal or perturbation-based evidence demonstrating that shared type 2 activity predicts or modifies disease activity across both organs (7, 11, 37, 38).
Neuroimmune signaling	Sensory-neuron activation or sensitization; IL-31-related signaling; neuropeptides; immune–neural communication; pruritus and sensory hyperresponsiveness. Representative readouts include IL-31, substance P, calcitonin gene-related peptide, itch or nasal-itch ratings, scratching or rubbing measures, and sensory-response measures.	AD: substantial evidence for IL-31-related pruritus and the itch–scratch cycle. AR: evidence for nasal sensory-afferent activation, neuropeptide signaling, itching, sneezing, and reflex secretory responses.	Direct pediatric AD–AR: Not established. Adult/mixed-age human: Supports nasal sensory-afferent, neuropeptide, and neurogenic inflammatory pathways. Animal/experimental: Supports IL-31-, neurokinin-, and substance P–mediated mechanisms. Pediatric single-organ AD/AR: Moderate-to-strong pediatric AD evidence; pediatric AR evidence is mainly clinical and symptom based.	Direct evidence that neuroimmune activation in one organ causes inflammatory activity or symptom changes in the other remains limited.	Narrative grade: Moderate Principal gap: Prospective assessment of whether neuroimmune measures predict synchronized or time-lagged skin and nasal activity (39–44).
Microbiome regulation	Skin, nasal, and anterior-nares microbiome composition and function; microbiome perturbation; microbial metabolites and products; functional metagenomic profiles; strain-level features when available; anterior-nares reservoir status, particularly *S. aureus* carriage; paired skin, nasal, and anterior-nares sampling.	AD: skin microbiome perturbation and *S. aureus*/pathobiont dominance are associated with barrier dysfunction, inflammation, pruritus, and severity. AR: altered nasal microbiome composition and function have been associated with mucosal inflammation and disease activity. Functional outputs may affect epithelial junctions, antimicrobial defense, alarmin release, innate immune tone, and epithelial-immune amplification.	Direct pediatric AD–AR: Emerging but insufficient; available cross-site findings are mainly cross-sectional. Adult/mixed-age human: Supports *S. aureus*–associated dysbiosis and altered nasal microbiome profiles. Animal/experimental: Supports microbial modulation of epithelial and immune pathways. Pediatric single-organ AD/AR: Strong pediatric AD evidence and growing pediatric AR evidence.	Direct evidence that anterior-nares reservoir status, microbial concordance, or functional/strain-level features mediate reproducible skin–nasal coupling in pediatric AD–AR comorbidity remains limited.	Narrative grade: Limited to moderate. Principal gap: longitudinal paired skin, nasal, and anterior-nares sampling with functional or strain-level profiling and control of local disease activity, infection, antibiotics, treatment, season, sampling site, and environmental exposure (45–51).

Evidence was appraised narratively according to source population, pediatric relevance, directness to pediatric AD–AR comorbidity, consistency across studies, and study design. The evidence categories indicate the relative level of support for each mechanistic domain and are not based on a validated grading system. Evidence from AD, AR, related allergic diseases, adult populations, or experimental models supports the biological plausibility of the proposed framework but should not be interpreted as direct validation of reproducible cross-organ coupling in children with AD–AR comorbidity. The listed components and readouts are illustrative research measures rather than validated biomarkers, diagnostic criteria, or treatment-selection tools.

## 6 Diagnostic assessment: current clinical practice, research biomarkers, and unvalidated network profiling

### 6.1 Current clinical assessment

Diagnosis of AD and AR should continue to follow established organ-specific clinical criteria. Clinical assessment should document onset, duration, recurrence, seasonality, potential triggers, treatment response, and functional consequences of both skin and nasal symptoms. For AD, the assessment should document lesion morphology and distribution, pruritus, scratching, sleep disturbance, and previous treatment responses. For AR, it should include sneezing, nasal itching, watery rhinorrhea, nasal obstruction, ocular symptoms, seasonal or exposure-related variation, and effects on sleep, school performance, and daily activities. Family history, relevant environmental exposures, and other atopic conditions should also be recorded. Temporal co-occurrence or parallel fluctuation of skin and nasal symptoms may be documented descriptively, but such observations do not by themselves establish mechanistic cross-organ coupling.

Physical examination should assess the skin and nasal cavity and disease severity should be evaluated with validated clinician-reported instruments such as the Eczema Area and Severity Index (EASI) or SCORing Atopic Dermatitis (SCORAD), whereas patient- or caregiver-reported symptom burden may be assessed using the Patient-Oriented Eczema Measure (POEM) and age-appropriate itch rating scales (52–55). AR may be characterized according to symptom duration and severity using the Allergic Rhinitis and its Impact on Asthma (ARIA) classification, together with instruments such as the Total Nasal Symptom Score (TNSS) or a visual analogue scale (VAS) when appropriate (56–59). These instruments are useful for quantifying organ-specific disease severity, symptom burden, and changes in disease control over time. However, these instruments do not directly measure cross-organ coupling or coordinated activity across the proposed mechanistic domains and should not be combined into an unvalidated composite “network score”.

Allergen sensitization testing should be performed when the clinical history suggests an IgE-mediated trigger and when the result is expected to influence diagnosis or management. Skin prick testing and serum allergen-specific IgE testing may help identify sensitization to clinically suspected allergens, whereas serum total IgE provides only nonspecific contextual information regarding atopic tendency (60). These test results should therefore be interpreted in conjunction with the clinical history and relevant exposure patterns.

Additional upper-airway investigations should be selected according to the clinical context. Nasal endoscopy may be considered when symptoms are atypical, persist despite appropriate treatment, or raise concern for structural obstruction, adenoidal disease, chronic rhinosinusitis, or another alternative diagnosis (61).

### 6.2 Research biomarkers

A range of candidate biomarkers may help investigate individual components of the proposed framework. Epithelial alarmins such as TSLP and IL-33 may provide information about epithelial stress and alarmin signaling (62). IL-31 may reflect pruritic neuroimmune activity; thymus and activation-regulated chemokine/CC chemokine ligand 17 (TARC/CCL17) may reflect type 2 inflammatory activity, particularly in AD; and neuropeptides such as substance P and CGRP may be relevant to neuroimmune signaling (62, 63).

Skin and nasal microbiome profiles and tissue- or fluid-based transcriptomic signatures may provide additional information regarding epithelial ecology, inflammatory pathways, and molecular heterogeneity. Nevertheless, their interpretation is affected by sampling site, age, disease activity, recent infection, treatment exposure, season, sequencing platform, analytic pipeline, and batch effects. Standardized sampling protocols, reproducible reference ranges, clinically meaningful thresholds, and prospective evidence of incremental utility are currently lacking. Candidate biomarkers and profiling approaches are summarized in **Table 3** according to a narrative categorization of their current clinical readiness. This categorization is intended to distinguish established clinical applications from research-stage and unvalidated approaches and should not be interpreted as a formally validated grading system.

**Table 3:** Current clinical tests, candidate biomarkers, and profiling approaches for pediatric AD–AR comorbidity.

Category	Test, biomarker, or profiling approach	Current role or proposed relevance	Clinical readiness and main limitations
Current clinical assessment	Serum total IgE	Nonspecific contextual information about IgE-associated atopic tendency.	Adjunctive clinical context only; low specificity and not a biomarker of cross-organ coupling (60).
Current clinical assessment	Allergen-specific IgE and skin prick testing	Identifies sensitization to clinically suspected allergens.	Established when clinically indicated; sensitization requires interpretation with history and exposure and does not measure cross-organ coupling (60).
Research biomarkers	TSLP and IL-33	Candidate indicators of epithelial stress, alarmin release, and upstream type 2 activation.	Research use only; no validated pediatric AD–AR-specific cutoff or sampling standard (62).
Research biomarkers	IL-31, substance P, and CGRP	Candidate indicators of pruritic neuroimmune activity and neurogenic inflammation.	Research/experimental use only; limited specificity, assay variability, and no validated pediatric AD–AR thresholds (42, 44, 62, 63).
Research biomarkers	TARC/CCL17	Candidate marker of type 2 chemokine activity and AD inflammatory severity.	Disease-specific adjunct or research biomarker; not validated for pediatric AD–AR comorbidity (62).
Research profiling	Skin, nasal, and anterior-nares microbiome profiles, including functional or strain-level features when available	May reflect cutaneous or upper-airway microbial ecology, anterior-nares reservoir status, *S. aureus* carriage, microbial functional outputs, and skin–nasal microbial concordance.	Exploratory research tool; affected by sampling site, treatment, infection, season, sequencing platform, analytic pipeline, and disease activity; causality and pediatric AD–AR-specific thresholds remain unvalidated (46–49).
Research profiling	Skin or nasal transcriptomic signatures	May identify epithelial, immune, neural, or allergen-related molecular pathways.	Discovery- and validation-stage research tool; high dimensionality, tissue specificity, cost, batch effects, and lack of external validation or clinical thresholds (62).

Established clinical tests support organ-specific assessment and evaluation of allergic sensitization. Candidate biomarkers and profiling approaches may help investigate the mechanistic domains and candidate cross-organ coupling in pediatric AD–AR comorbidity, but are unvalidated for routine diagnosis, prognosis, treatment selection, or cross-organ coupling measurement. Clinical-readiness categories are narrative summaries, not a validated grading scale.

### 6.3 Unvalidated network profiling

Integrated profiling could theoretically combine conventional clinical variables with allergen sensitization, epithelial and immune mediators, neuroimmune symptoms, microbiome features, and transcriptomic or other omics data using network-based analytical methods. Composite models derived from these domains might eventually help identify biologically distinct phenotypes or estimate the risk of disease persistence, cross-organ involvement, or treatment response.

Clinical translation would require prospective studies with repeated measurements, comparison against organ-specific models, independent validation, and evidence of discrimination, calibration, reproducibility, feasibility, and incremental clinical utility across relevant pediatric subgroups.

## 7 Management of pediatric AD–AR comorbidity: guideline-based care and research implications

The management of pediatric AD–AR comorbidity should remain grounded in established, age-appropriate guidelines for AD and AR (64, 65). Its management relevance is to support coordinated assessment of combined burden and to frame research questions about whether shared pathways influence cross-organ coupling or treatment responses. Guideline-based organ-specific care, selected disease-specific agents with mechanistic relevance, and research-stage interventions are summarized in **Table 4**.

**Table 4:** Treatment approaches relevant to pediatric AD–AR comorbidity: mechanisms, pediatric evidence, and safety considerations.

Category	Intervention	Mechanism or therapeutic role	Pediatric evidence and disease context	Evidence/status in pediatric AD–AR comorbidity	Major safety and monitoring considerations
Guideline-based organ-specific care	Emollients and barrier care	Reduce transepidermal water loss, support epidermal barrier recovery, and reduce exposure to clinically relevant irritants.	Established across pediatric AD care; benefits are primarily organ-specific and formulation-dependent.	Core AD management; no evidence that barrier care alone modifies AR or cross-organ coupling.	Generally well tolerated; select age-appropriate formulations and monitor for irritation or contact allergy. Avoid unnecessary fragranced or sensitizing ingredients (64, 67).
Guideline-based organ-specific care	Topical corticosteroids and topical calcineurin inhibitors	Suppress cutaneous inflammation and pruritus; topical calcineurin inhibitors provide steroid-sparing anti-inflammatory treatment at selected sites.	Strong pediatric AD evidence and guideline support across age- and site-specific treatment plans.	Established for AD; not validated as a treatment for pediatric AD–AR comorbidity or AR.	Use corticosteroid potency, site, and duration appropriately; excessive or prolonged use may cause local adverse effects. Topical calcineurin inhibitors may cause transient burning or stinging (64, 67).
Guideline-based organ-specific care	Intranasal corticosteroids	Reduce nasal mucosal inflammation and improve congestion, rhinorrhea, sneezing, and itching.	Established pediatric AR evidence; selection depends on age, severity, and product labeling.	Core AR treatment; no direct evidence that nasal control modifies the course of AD.	Local irritation and epistaxis may occur; correct spray technique is important. Consider cumulative corticosteroid exposure and growth monitoring when prolonged treatment is required (65, 68).
Guideline-based organ-specific care	Second-generation antihistamines	H1-receptor blockade reduces histamine-mediated symptoms.	Commonly used in pediatric AR; oral second-generation agents are preferred to first-generation sedating antihistamines.	Symptom-directed AR management; no direct evidence for disease modification or cross-organ benefit.	Sedation varies among antihistamines; first-generation agents may impair attention and should generally be avoided (65, 68).
Guideline-based organ-specific care	Allergen immunotherapy	Induces allergen-specific immune tolerance through repeated administration of clinically relevant inhalant allergens.	Pediatric evidence supports use in appropriately selected AR/rhinoconjunctivitis patients with confirmed clinically relevant sensitization.	Established for selected AR patients; not validated as a treatment for pediatric AD–AR comorbidity as a unified entity.	Local reactions are common; systemic reactions, including anaphylaxis, can occur. Patient selection, trained supervision, emergency preparedness, and adherence are essential (69).
Agents with mechanistic relevance and disease-specific evidence	Dupilumab	Blocks IL-4Rα-mediated IL-4 and IL-13 signaling, affecting type 2 inflammation, pruritus, and epithelial barrier dysfunction.	Randomized trials demonstrate improvement in skin lesions and pruritus in infants, children, and adolescents with moderate-to-severe AD.	Established for age-eligible moderate-to-severe AD; not established specifically for pediatric AD–AR comorbidity or uncomplicated AR.	Monitor for hypersensitivity, conjunctivitis/keratitis, eosinophilic conditions, helminth infection, and vaccination considerations; approved ages and indications vary by jurisdiction (70–74).
Agents with mechanistic relevance and disease-specific evidence	Omalizumab	Binds free IgE and reduces FcϵRI-mediated mast-cell and basophil activation.	Pediatric efficacy is established in allergic asthma. Seasonal AR evidence includes pediatric studies, often involving adjunctive use with allergen immunotherapy, as well as mixed adolescent–adult trials.	Not established for pediatric AD–AR comorbidity; evidence from asthma or AR alone cannot establish comorbidity efficacy.	Boxed warning for anaphylaxis; initiate with appropriate observation and emergency preparedness. Injection-site reactions and other label-specific precautions should be considered (75–79).
Agents with mechanistic relevance and disease-specific evidence	Tezepelumab (anti-TSLP)	Blocks TSLP, an epithelial alarmin involved in initiating and amplifying type 2 inflammation.	Pediatric evidence is available in severe asthma; evidence in upper-airway disease derives from CRSwNP and is not specific to uncomplicated AR.	No established role in AD, uncomplicated AR, or pediatric AD–AR comorbidity.	Hypersensitivity, helminth infection, and vaccination considerations; avoid live attenuated vaccines and follow indication-specific labeling (80–83).
Agents with mechanistic relevance and disease-specific evidence	Nemolizumab	Blocks IL-31 receptor α signaling, targeting pruritus and neuroimmune signaling in AD.	Phase 3 trials show improvement in inflammation and pruritus in adolescents and adults with moderate-to-severe AD.	Established for age-eligible AD in some jurisdictions; no evidence of efficacy for AR or cross-organ benefit in pediatric AD–AR comorbidity.	Monitor for hypersensitivity and vaccination considerations; age eligibility and approved indications are jurisdiction-specific (40, 84).
Agents with mechanistic relevance and disease-specific evidence	Oral JAK inhibitors: upadacitinib and abrocitinib	Inhibit JAK1-dependent intracellular signaling used by multiple cytokines relevant to AD, including IL-4, IL-13, and IL-31.	Randomized studies demonstrate efficacy in adolescents with moderate-to-severe AD, including improvement in skin lesions and pruritus.	Established benefit is disease-specific to AD; efficacy for AR or pediatric AD–AR comorbidity has not been demonstrated.	Important warnings include serious infection, mortality, malignancy, major adverse cardiovascular events, thrombosis, and hematologic, hepatic, and lipid abnormalities. Pretreatment screening and laboratory monitoring are product-specific (85–89).
Research-stage interventions	Investigational IL-33–targeted agents	Target IL-33-dependent epithelial alarm and type 2 inflammatory signaling.	Early clinical evaluation has been conducted primarily in adults with AD; pediatric evidence is lacking.	Investigational; no established role in pediatric AD–AR comorbidity.	Long-term pediatric safety, dosing, and monitoring requirements have not been established (90, 91).
Research-stage interventions	Neurokinin receptor antagonists and other investigational neuroimmune approaches	Aim to modulate neuropeptide signaling, pruritus, neurogenic inflammation, or sensory amplification.	Limited and inconsistent evidence, primarily from adult or mixed-age AD studies; pediatric data are sparse.	Investigational; no validated role in pediatric AD–AR comorbidity.	Safety is agent-specific and incompletely characterized for pediatric use; efficacy and risk–benefit require prospective validation (42, 44, 92, 93).
Research-stage interventions	Microbiome-directed interventions	Probiotics, synbiotics, topical commensal bacteria, and live biotherapeutic products aim to modify epithelial–microbial–immune homeostasis.	Pediatric AD and AR studies report heterogeneous, product-specific findings; topical commensal preparations remain early-phase.	Investigational; no microbiome-directed intervention has been validated specifically for pediatric AD–AR comorbidity.	Strain and product identity, contamination control, manufacturing consistency, infection risk, dosing, and long-term safety require careful evaluation; routine use is not established (94–97).

This table organizes guideline-based organ-specific care, agents with mechanistic relevance, and research-stage interventions relevant to pediatric AD–AR comorbidity.

It is not a treatment algorithm and does not imply approval for pediatric AD–AR comorbidity as a unified entity.

### 7.1 Principles of coordinated, guideline-based management

The severity and control of each disease should be assessed separately, while the management plan should consider the combined effects of pruritus, nasal obstruction, sleep disturbance, treatment burden, and health-related quality of life.

Patient and caregiver education is an integral component of management (66). Education should address chronic and relapsing disease, realistic goals, correct topical and intranasal medication use, adherence, recognition of exacerbations, and the distinction between maintenance therapy and short-term adjustments.

### 7.2 Foundational and organ-specific therapies

For AD, regular emollient use, reduction of clinically relevant irritants, and appropriate topical anti-inflammatory treatment remain central. Topical corticosteroids and topical calcineurin inhibitors should be used according to established recommendations for age, severity, anatomical site, treatment phase, and individual patient characteristics (64, 67).

For AR, intranasal corticosteroids, second-generation antihistamines, saline irrigation, reduction of clinically relevant allergens, and allergen immunotherapy for selected patients remain evidence-based options (65, 68, 69).

These treatments target established organ-specific processes; whether control of one disease consistently modifies the clinical course of the other remains unproven in pediatric AD–AR comorbidity.

### 7.3 Selected biologic and small-molecule agents: mechanistic relevance and evidence boundaries

Several biologic and small-molecule agents approved or investigated for AD, AR, or other defined type 2 inflammatory diseases have molecular targets that overlap with pathways discussed within the proposed systemic epithelial–immune–neural framework. Evidence remains disease-specific, and findings from AD, AR or allergic rhinoconjunctivitis, asthma, or chronic rhinosinusitis with nasal polyps should not be interpreted as direct evidence for a unified pediatric AD–AR indication. **Table 4** summarizes mechanisms, pediatric evidence, disease-specific contexts, and safety considerations.

#### 7.3.1 Biologic agents

Dupilumab inhibits IL-4 and IL-13 signaling and has established efficacy in age-eligible children with moderate-to-severe AD (70–74). It may provide an opportunity to examine whether systemic type 2 pathway modulation initiated for an established AD indication is associated with prespecified nasal outcomes or altered temporal coupling. Such findings would require the causal safeguards described in Section 2.4 and would not, by themselves, establish skin-to-nasal mediation.

Omalizumab targets IgE-dependent effector responses and has established pediatric evidence in allergic asthma, with additional evidence in seasonal AR, including studies involving adjunctive allergen immunotherapy (75–79). These findings support future studies in sensitization-defined subgroups but do not establish efficacy for pediatric AD–AR comorbidity as a unified condition.

Tezepelumab targets TSLP and is relevant to the epithelial alarmin domain of the framework (80, 81). Pediatric evidence is mainly from severe asthma, and upper-airway evidence mainly from chronic rhinosinusitis with nasal polyps rather than uncomplicated AR (82, 83); its role here remains hypothesis-generating.

Nemolizumab targets IL-31 receptor signaling and has demonstrated efficacy for inflammation and pruritus in adolescents and adults with AD (40, 84). It may help investigate the neuroimmune domain, but AR efficacy or cross-organ benefit in pediatric AD–AR comorbidity has not been established.

#### 7.3.2 Oral JAK inhibitors

Upadacitinib and abrocitinib inhibit Janus kinase 1 (JAK1)-dependent signaling used by several cytokines relevant to AD and have demonstrated efficacy in adolescents with moderate-to-severe AD (85–89). Their effects on inflammatory and pruritic pathways may be informative for mechanistic research, but no AR efficacy or cross-organ benefit has been established. Because these agents carry important regulatory warnings and require product-specific screening and monitoring, they should be used only for approved disease-specific indications under appropriate clinical supervision.

### 7.4 Research priorities for emerging interventions

Investigational IL-33–targeted agents represent another area for future study. IL-33 is involved in epithelial alarmin signaling and type 2 inflammation, but early clinical evaluation has primarily been conducted in adults with AD, and pediatric evidence remains lacking (90, 91). Neuroimmune approaches, including neurokinin receptor antagonism, remain research priorities because they may influence pruritus, scratching, sleep, and symptom amplification, although evidence is limited or inconsistent and largely not pediatric AD–AR-specific (42, 44, 92, 93).

Microbiome-directed interventions have been studied separately in pediatric AD and AR, but probiotic and synbiotic findings are heterogeneous and topical commensal or live biotherapeutic approaches remain early phase (94–97). No microbiome-directed intervention has been validated for pediatric AD–AR comorbidity.

Future studies should enroll well-characterized children with AD–AR comorbidity and prespecify organ-specific outcomes, system-level burden, and safety outcomes. This design may help determine whether emerging interventions affect only an individual disease domain or are associated with broader changes in pediatric AD–AR burden.

## 8 Discussion

### 8.1 Conceptual contribution and interpretation of the evidence

This article proposes the systemic epithelial–immune–neural framework as a hypothesis-driven model for investigating whether skin and nasal disease activity in pediatric AD–AR comorbidity show reproducible cross-organ coupling after shared causes and contextual factors have been considered. Its contribution is organizational rather than the identification of new mechanisms: it links epithelial barrier and alarmin signaling, type 2 immune activation, neuroimmune signaling, and microbiome regulation to an operational definition, directed relationships, observable consequences, testable predictions, and falsification criteria.

Current evidence supports biological plausibility but remains uneven. Epidemiological studies establish frequent co-occurrence and related trajectories, whereas mechanistic evidence is strongest for epithelial, immune, neural, and microbiome-related processes within AD or AR considered separately. Shared epithelial alarmin and type 2 immune pathways support substantial mechanistic overlap; proposed neuroimmune and microbiome-related cross-organ roles rely more heavily on indirect evidence. Thus, overlapping biomarkers, synchronous symptoms, sequential onset, or concurrent improvement during treatment may be compatible with the framework but cannot alone establish cross-organ coupling.

### 8.2 Clinical implications and current boundaries

The immediate clinical implication is coordinated assessment of the combined burden of pediatric AD–AR comorbidity. Clinical evaluation should remain based on age-appropriate history, examination, validated organ-specific severity and control measures, and allergen sensitization testing when indicated. Temporal skin–nasal symptom patterns may be documented as descriptive clinical information or research variables, but not as evidence of direct cross-organ coupling.

### 8.3 Limitations and a testable research agenda

This article is a theory-informed narrative synthesis rather than a systematic review or formal causal analysis. The supporting literature varies in age, disease definitions, sampling methods, treatment exposure, follow-up, outcomes, and confounding adjustment; much evidence also derives from AD or AR studied separately, other allergic diseases, adult or mixed-age cohorts, or experimental models. These limitations mean that the proposed domains, configurations, evidence summaries, and research readouts should remain provisional and revisable.

Future research should test whether skin and nasal disease activity show residual, time-ordered coupling after shared determinants have been addressed. Prospective pediatric cohorts should combine repeated organ-specific assessment, paired biological sampling where feasible, and documentation of treatment, adherence, infection, exposure, season, and baseline severity. Candidate epithelial, immune, neuroimmune, and microbiome-related measures should then be evaluated for mediation, effect modification, and incremental predictive value. The framework will be strengthened only if residual skin–nasal coupling, biological mediation, reproducible subgroups, or cross-organ responses to ethical perturbation are independently replicated and add information beyond simpler organ-specific models.

## 9 Conclusion

Pediatric AD–AR comorbidity raises a central question: whether skin and nasal disease activity are measurably coupled beyond shared susceptibility and parallel organ-specific processes. The systemic epithelial–immune–neural framework translates this question into an operational and falsifiable research model by linking epithelial barrier and alarmin signaling, type 2 immune activation, neuroimmune signaling, and microbiome regulation to directed relationships, testable predictions, and falsification criteria.

Current evidence supports the biological plausibility of the framework but does not establish reproducible skin–nasal coupling in children. The framework should therefore guide research rather than diagnosis or treatment selection. Future prospective studies should combine repeated skin and nasal evaluation, paired biological sampling, careful control of shared determinants, and ethically appropriate mechanistic perturbation to determine whether cross-organ coupling can be reproduced, which children exhibit it, and whether it adds clinically meaningful information beyond conventional organ-specific assessment.

## Data Availability

The original contributions presented in the study are included in the article/supplementary material. Further inquiries can be directed to the corresponding author.
